# Supplementary material for: HIVprotI: an integrated web based platform for prediction and design of HIV proteins inhibitors
Source: J Cheminform. 2018 Mar 9;10:12. doi: 10.1186/s13321-018-0266-y (PMC5845081; doi:10.1186/s13321-018-0266-y)
Supplement: Supplementary file 2 — Additional file 2. Source code of HIVProtI web server. [file 13321_2018_266_MOESM2_ESM.zip › HIVprotI_Source-code/hivprotI/weka-3-6-6/doc/weka/classifiers/functions/SMOreg.html]

SMOreg


---


|  |  |  |
| --- | --- | --- |
| **Overview** \| **Package** \| **Class** \| **Tree** \| **Deprecated** \| **Index** \| **Help** \| **Weka's home** | |  |
| **PREV CLASS**   **NEXT CLASS** | **FRAMES**    **NO FRAMES**     **All Classes** |  |
| SUMMARY: NESTED \| FIELD \| CONSTR \| METHOD | DETAIL: FIELD \| CONSTR \| METHOD |  |


---


## weka.classifiers.functions Class SMOreg

```
java.lang.Object
  weka.classifiers.Classifier
      weka.classifiers.functions.SMOreg
```

**All Implemented Interfaces:**: java.io.Serializable, java.lang.Cloneable, AdditionalMeasureProducer, CapabilitiesHandler, OptionHandler, RevisionHandler, TechnicalInformationHandler, WeightedInstancesHandler

---

``` public class SMOreg extends Classifier implements WeightedInstancesHandler, AdditionalMeasureProducer, TechnicalInformationHandler ```

SMOreg implements the support vector machine for regression. The parameters can be learned using various algorithms. The algorithm is selected by setting the RegOptimizer. The most popular algorithm (RegSMOImproved) is due to Shevade, Keerthi et al and this is the default RegOptimizer.
